# Can bronchoconstriction and bronchodilatation in horses be detected using electrical impedance tomography?

**DOI:** 10.1111/jvim.16152

**Published:** 2021-05-11

**Authors:** Cristy Secombe, Andy Adler, Giselle Hosgood, Anthea Raisis, Martina Mosing

**Affiliations:** ^1^ School of Veterinary Medicine, Murdoch University Perth Australia; ^2^ Systems and Computer Engineering, Carleton University Ottawa Canada

**Keywords:** albuterol, equine, equine asthma, flow volume loop, flowmetric plethysmography, pulmonary function test

## Abstract

**Background:**

Electrical impedance tomography (EIT) generates images of the lungs based on impedance change and was able to detect changes in airflow after histamine challenge in horses.

**Objectives:**

To confirm that EIT can detect histamine‐provoked changes in airflow and subsequent drug‐induced bronchodilatation. Novel EIT flow variables were developed and examined for changes in airflow.

**Methods:**

Bronchoconstriction was induced using stepwise histamine bronchoprovocation in 17 healthy sedated horses. The EIT variables were recorded at baseline, after saline nebulization (control), at the histamine concentration causing bronchoconstriction (C_max_) and 2 and 10 minutes after albuterol (salbutamol) administration. Peak global inspiratory (PIF_EIT_) and peak expiratory EIT (PEF_EIT_) flow, slope of the global expiratory flow‐volume curve (FV_slope_), steepest FV_slope_ over all pixels in the lung field, total impedance change (surrogate for tidal volume; VT_EIT_) and intercept on the expiratory FV curve normalized to VT_EIT_ (FV_intercept_/VT_EIT_) were indexed to baseline and analyzed for a difference from the control, at C_max_, 2 and 10 minutes after albuterol. Multiple linear regression explored the explanation of the variance of Δflow, a validated variable to evaluate bronchoconstriction using all EIT variables.

**Results:**

At C_max_, PIF_EIT_, PEF_EIT_, and FV_slope_ significantly increased whereas FV_intercept_/VT decreased. All variables returned to baseline 10 minutes after albuterol. The VT_EIT_ did not change. Multivariable investigation suggested 51% of Δflow variance was explained by a combination of PIF_EIT_ and PEF_EIT_.

**Conclusions and Clinical Importance:**

Changes in airflow during histamine challenge and subsequent albuterol administration could be detected by various EIT flow volume variables.

**Abbreviations**
*A*_10min_
: 10  minutes post albuterol administration
*A*_2min_
: 2  minutes post albuterol administration
AU: arbitrary units
C_max_
: histamine concentration causing maximal bronchoconstriction
EIT: electrical impedance tomography
FP: flowmetric plethysmography
FV_intercept_/VT_EIT_
: the intercept between the steep and the horizontal part of the expiratory flow volume EIT loop, and given as the ratio between intercept to VT_EIT_
FV_slope_
: global steepness of the slope of the initial expired volume of the expiratory EIT FV loop
FV_slope_max: the value of the steepest slope of all the FV loops generated within the ROI
PEF_EIT_
: EIT peak global expiratory flow
PEF_Spiro_
: pneumotachograph peak expiratory flow
PIF_EIT_
: EIT peak global inspiratory flow
PIF_Spiro_
: pneumotachograph peak inspiratory flow
RIP: respiratory inductance plethysmography
ROI: region of interest
VT_EIT_
: EIT tidal volume
VT_Spiro_
: pneumotachograph tidal volume
Δflow: validated FP variable
Δflow: validated FP variable

## 1 INTRODUCTION

Mild to moderate asthma in horses commonly is diagnosed based on clinical signs, increased tracheal mucus viewed on endoscopy, and bronchoalveolar lavage cytology.^1^, ^2^ It is unclear which diagnostic tool is most appropriate because a clinically applicable objective lung function test is not readily available. The lack of alignment in diagnostic approach between practitioners and international consensus presents a barrier to advancing evidence‐based practice^2^, ^3^ and there is a need for a sensitive diagnostic tool, ideally accessible and noninvasive, that can provide an objective measurement of lung function in the field. Such devices would best detect subtle to marked alteration in lung mechanics, which occurs across the range of manifestations seen in the stages of asthma in horses.^1^

Electrical impedance tomography (EIT) is a noninvasive, radiation‐free functional imaging modality, which allows assessment of ventilation of conscious and anesthetized horses.^4^, ^5^, ^6^ The fundamental principle of EIT is Ohm's law which describes the resistance of tissue to electrical current from measurements of voltage. Alternating currents are used instead of direct current making impedance the main outcome measure for EIT as the analogue to resistance. Lung tissue impedance changes during breathing as a function of air content. Electrodes equidistantly mounted on a belt are placed around the thorax. Weak alternating currents are applied and voltages measured between pairs of electrodes. From these measurements, changes in impedance within the thorax can be calculated and cross‐sectional images of the distribution of impedance changes within the thorax are displayed 50 times per second. Based on the reported linear relationship between EIT impedance change and changes in lung air volume, it can be assumed that impedance change correlates with gas volume, and total impedance change over a single breath can be used as a surrogate for tidal volume.^7^, ^8^, ^9^ Therefore, peak inspiratory (PIF_EIT_) and peak expiratory (PEF_EIT_) global flow can be evaluated by calculating the first derivative of the global EIT volume signal.^10^ Electrical impedance tomography derived flow signals have been shown to detect changes in airflow during histamine bronchoprovocation in horses.^11^ In that pilot study, flowmetric plethysmography (FP) described by the variable delta flow (Δflow) was compared against regional and global peak inspiratory and expiratory flow variables calculated from the EIT measurements. Although EIT flow variables and Δflow represent different measurement principles of flow, EIT flow variables were shown to follow the same pattern of change as Δflow over the duration of the histamine challenge. The authors concluded that EIT has potential as a noninvasive, objective diagnostic tool to monitor airflow changes occurring in horses with asthma.

Our aim was to confirm the observation noted for EIT flow variables in the pilot study by examining histamine‐provoked changes in airflow in a larger population of horses and to determine if novel EIT variables could provide additional information on changes in breathing pattern during bronchoconstriction, as well as evaluate subsequent airflow changes after drug‐induced bronchodilatation. A secondary aim was to compare EIT flow variables with the corresponding flow variables measured using a pneumotachograph and the validated FP variable Δflow.

It was hypothesized that all EIT‐derived variables would change during histamine challenge and return to preprovocation levels after drug‐induced bronchodilatation. Secondarily, it was hypothesized that EIT flow variables would follow the same pattern as standard flow variables and Δflow, and finally that the variance in Δflow could be best explained by the EIT flow variables.

## 2 MATERIALS AND METHODS

### 2.1 Animals

Twenty horses (12 Standardbreds and 8 Thoroughbreds) from the institutional teaching herd were included. The horses included 3 mares and 17 geldings, with a mean (SD) age of 11.7 (7.9) years and body weight of 511 (64) kg. The median (range) body condition score was 6 of 9 (range, 5‐7/9).^12^ Horses were considered clinically healthy based on clinical examination and long‐term history. Before the study, the horses were housed in an irrigated paddock and received supplemental feed of meadow hay.

### 2.2 Preparation

Each horse was walked into stocks and had an 18G catheter (Surflo, Terumo, Philippines) aseptically placed in the right jugular vein. The horse then was sedated with a loading dose of 5 μg/kg of detomidine (Detomo Vet, Ceva, Glenorie, NSW, Australia) IV before immediately receiving a detomidine constant rate infusion (12.5 μg/kg/hour) administered through a fluid pump. The hair over the thorax directly caudal to the scapula (5th‐6th intercostal space) was moistened with water and electrically nonconductive ultrasound gel was applied circumferentially in this region. Thereafter, a stretchable neoprene EIT belt with 32 equidistantly‐mounted stainless steel electrodes was placed under slight tension around the thorax on top of the gel. Contact with the skin was maintained by application of a cohesive stretchable bandage placed over the EIT belt. The belt was connected to the EIT device (BBvet, SenTec AG, Landquart, Switzerland). Sufficient contact of the electrodes with the skin was verified by the EIT software and visual inspection of adequate electrode‐skin resistance values during the study period. The EIT measurements were performed with 47 impedance tomograms per second. A modified Graz consensus reconstruction algorithm for EIT (GREIT)^13^ adapted for horse anatomy was used to generate EIT images for each horse, representing breathing‐related regional changes of impedance. Additional details on the use of EIT technology and image reconstruction are reported in the literature.^13^, ^14^, ^15^, ^16^

Each horse was concurrently fitted with FP hardware to measure the effect of histamine bronchoprovocation on Δflow (Open Plethysmography, Ambulatory Monitoring, Ardsley, New York). A facemask, pneumotachograph (No. 5 Fleisch), and abdominal and thoracic respiratory inductance plethysmography bands (RIP), which make up the FP unit, were fitted to the horse and calibrated according to the manufacturer's instructions.^17^ Head carriage was maintained on a stand in a consistent horizontal position to avoid nasal edema.^18^

### 2.3 Histamine bronchoprovocation

Data collection commenced 10 minutes after the initiation of the detomidine constant rate infusion and fitting of all devices. Data was collected in each animal before treatment (baseline), after nebulization (Pari LC Plus reusable nebulizer, Pari Respiratory Equipment, Midlothian, Virginia) of saline alone (control), after nebulization of histamine diphosphate in saline solution in increasing concentrations until predefined endpoints were reached. Each solution was nebulized for 2 minutes. At each examination point, lung function data was recorded for 3 minutes using the EIT, spirometry and the FP system simultaneously.

Saline (0.9%) was nebulized as a control for reaction to diluent followed by series of increasing doses of histamine diphosphate in saline solution, starting with 4 mg/mL (4, 8, 16, 32 mg/mL) until at least 1 of the following 2 criteria was reached: (a) observation of subjective changes in breathing pattern corresponding to changes in airway diameter (increase in abdominal expiratory effort and respiratory rate) and an objective increase of Δflow of 50% over the control response, or (b) a maximum concentration of 32 mg/mL of histamine diphosphate was reached according to the manufacturer's instructions.^17^ This time point was referred to as C_max_.

After reaching 1 of the 2 described end points and data collection for C_max_, albuterol sulfate (Ventolin, GlaxoSmithKline, Victoria, Australia) was administered into the face mask using a metered dose inhaler (100 μg/actuation to ideally dose at 2 μg/kg). One puff was administered via the nebulizer port of the face mask at the beginning of each inspiration until the predefined dose was reached. Data collection for 3 minutes started 2 minutes (*A*_2min_) and 10 minutes (*A*_10min_) after the last puff was administered. Once data collection for *A*_2min_ and *A*_10min_ was complete, all instruments and devices were removed, the detomidine CRI stopped, and the horse was observed for an additional 10 minutes in the stocks before being returned to the paddock.

### 2.4 Electrical impedance tomography variables

A sequence of at least 8 and a maximum of 22 breaths free of any movement artifacts was preselected for each time point depending on the respiratory rate and cooperation of the horse during the 3‐minute measurement period using a dedicated software (Ibex, SenTec AG, Landquart, Switzerland).^19^ Breaths with obvious movement artifacts were removed manually. The EIT signal of each preselected sequence was exported and analyzed using custom software,^20^ which is available as part of EIDORS.^21^ The following variables were derived from the EIT signal:

#### 2.4.1 Gas flow variables and EIT‐derived tidal volume

**FIGURE 1:**
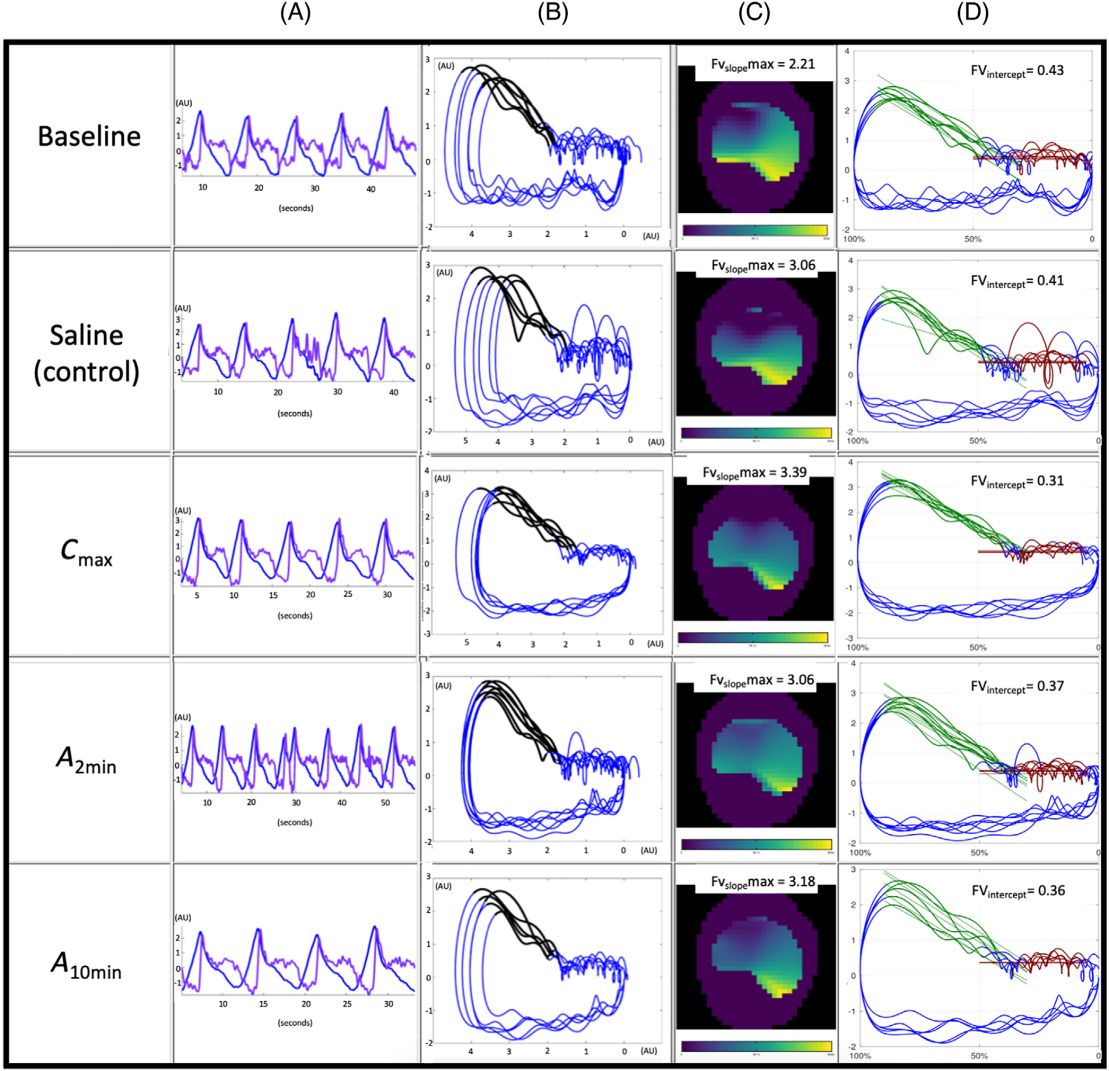
Electrical impedance tomography (EIT) measurements from 1 horse at baseline, after saline nebulization as control, at maximum bronchoconstriction (C_max_) and 2 (*A*_2min_) and 10 (*A*_10min_) after albuterol administration. A, The impedance curve measured by EIT representing the volume signal in blue and the corresponding flow signal in purple calculated as the first derivative from the volume signal plotted against time. Volume and flow are measured in EIT arbitrary units (AU). B, The global flow volume (FV) loop obtained from the EIT flow and volume signal and the FV slope in early expiration marked in black. C, Pictorial representation of the steepness of slopes of FV loop for each pixel within the lung regions and the value of the steepest slope (lightest color) in all FV loops generated within the lung area (FV_slope_max). D, The EIT FV loop duplicated on a grid to determine a novel variable of the expiratory component of the FV loop namely the FV intercept as the ratio between intercept to EIT derived tidal volume (FV_intercept_/VT_EIT_) (Figure 2)

The global impedance changes, representing the EIT volume signal in arbitrary units (AU), was calculated as the sum of all pixels within the entire EIT image. The tidal volume‐related EIT factor VT_EIT_ was calculated by subtracting the impedance distribution at start of inspiration from the impedance distribution at end of inspiration (Figure 1A).^7^, ^8^, ^9^ From the EIT volume signal, the global EIT gas flow was calculated as the first derivative of the global volume over each breath (Figure 1A).^10^ From the flow signal, global peak inspiratory (PIF_EIT_) and global peak expiratory (PEF_EIT_) flow were calculated for the whole lung for each breath.^11^

#### 2.4.2 Flow‐volume loop variables

From the obtained flow and volume signal, a global EIT flow‐volume (FV) loop (Figure 1B) and a FV loop for each pixel within the lung region of interest (ROI) were constructed. The FV loops were visually inspected to determine if a biphasic pattern could be identified. The slope of the expiratory portion of the FV loop was determined by the software algorithm. For each breath, the first part after peak expiration was analyzed and the slope of the best‐fit line taken (black aspect of FV loop; Figure 1B). The FV slope was the averaged over all measured breaths. The steepness of slopes of each pixel FV loop within the ROI was calculated and the value of the steepest slope in all FV loops generated within the lung area was identified (FV_slope_max; Figure 1C).

**FIGURE 2:**
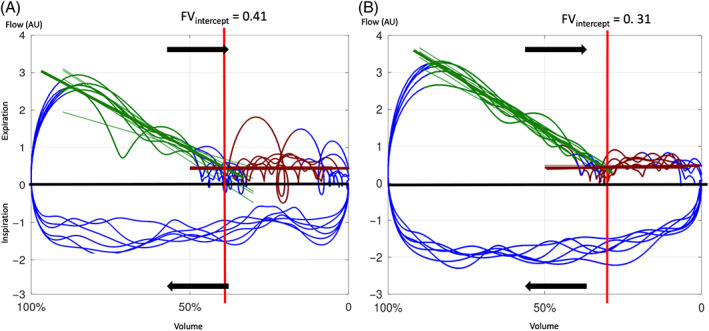
Graphical illustration of flow volume loops generated from 6 breaths during the measurements at baseline (A) and C_max_ (B) in the same horse as in Figure 1 and determination of the intercept in the FV loop (FV_intercept_/VT_EIT_). The volume change during inspiration and expiration are indicated by arrows. The expiratory portion of the curve is depicted by the portion of the loop with positive flow. The volume increase during inspiration is plotted toward the left on the x‐axis. The computing of the FV intercept slopes and lines are depicted in green and brown. The FV intercept is indicated by a red line and is the percentage of the expiratory portion of the flow volume loop where flow is constant

A distinct change in flow during expiration was observed, where the flow became constant over the final proportion of expiration. A novel variable was defined, determined by manual measurement of the intercept between the steep and the horizontal part of the expiratory FV loop. The mean intercept of all FV loops is given as the ratio between intercept to VT_EIT_ (FV_intercept_/VT_EIT_; Figures 2 and 1D).

### 2.5 Spirometry and flowmetric plethysmography variables

Peak inspiratory (PIF_Spiro_), peak expiratory (PEF_Spiro_) flows and tidal volume (VT_Spiro_) were measured using a calibrated Fleisch pneumotachograph. The commercial FP system (Open Plethysmography, Ambulatory Monitoring, Ardsley, New York) utilized software to derive a flow factor, Δflow, which was calculated based on spirometry and respiratory inductance band measurements by FP software. This flow factor measures the maximum difference between the flow derived from the pneumotachograph and the calibrated flow from the respiratory inductance bands.^17^ Delta flow has been validated as a variable that can detect narrowing in airway diameter after histamine bronchoprovocation.^18^, ^22^ The same software was used to record the spirometry data measured by the pneumotachograph.

### 2.6 Data analysis

For each variable, an average was calculated across all breaths at each time point for each horse. This value was the response of interest for statistical analysis. All variables at each examination point were indexed for each horse as a fraction of the baseline (*y*_i_ = *C*_i_/BL − 1); where *C*_i_ is the value measured at administration of saline (diluent control), histamine concentration when bronchoprovocation was deemed to have occurred (C_max_), or administration of albuterol at 2 minutes or 10 minutes post‐administration. Each index was verified to follow a normal distribution at each examination point by inspection of Q‐Q plots and failure to reject the null hypothesis of normality using the Shapiro‐Wilk statistic at *P* < .05.

Indexed variables for examination points beyond baseline were evaluated for a difference at each intervention (time) using a mixed linear model accounting for repeated measurements. A compound symmetry covariance structure was used, which assumes that the correlation between 2 measurements is the same, regardless of how far apart they are. Least squares means at each examination point were generated and compared to the null hypothesis of the diluent control (saline) value for all indexed variables. All analyses were considered significant at a Dunnett‐adjusted *P* ≤ .05.

To explore the explanation of the variance of Δflow, multiple linear regression was used with all EIT variables entered into the model and the best variable subset chosen based on adjusted *R* ^2^ and Mallow's p (Cp). Commercial software (SAS v 9.4, SAS Institute, Cary, North Carolina) was used for the analysis.

## 3 RESULTS

**FIGURE 3:**
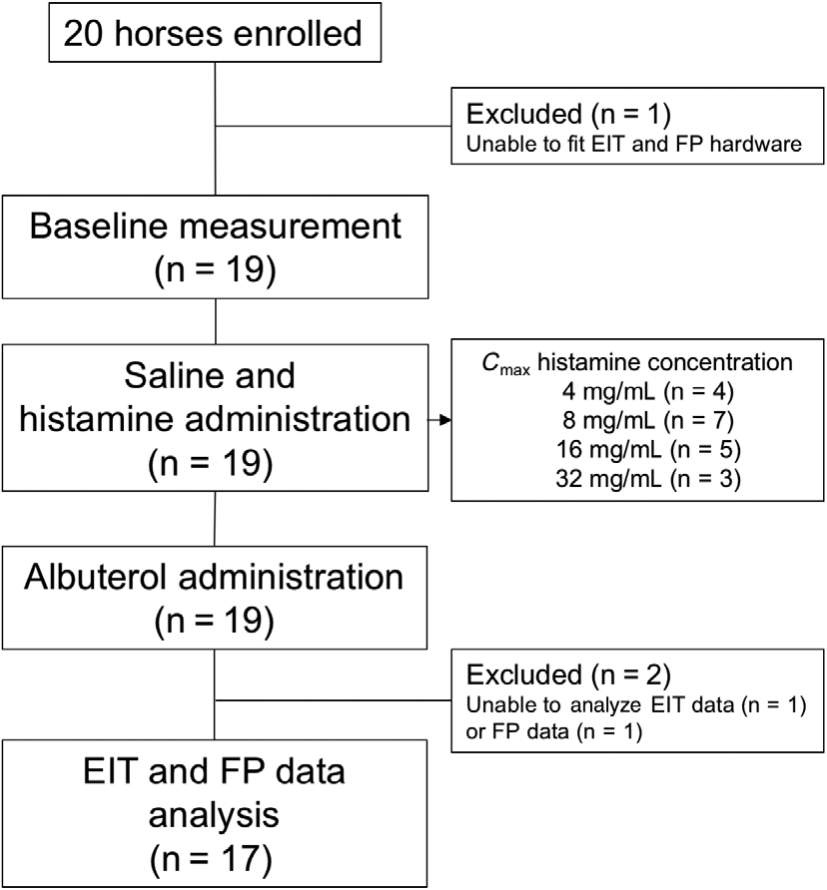
Flow diagram documenting the experimental timeline and exclusions. C_max_, histamine concentration causing maximal bronchoconstriction; EIT, electrical impedance tomography; FP, flowmetric plethysmography

Twenty horses were enrolled in the study (Figure 3), 19 horses allowed instrumentation of both the EIT and FP hardware and accepted subsequent histamine bronchoprovocation and recovered as expected. All horses received the diluent control nebulization (saline), and the endpoints of histamine provocation (C_max_) were reached at various concentrations of histamine (Figure 3). On examination of the data posttesting, FP data could not be used in 1 horse because the RIP bands ceased to register a signal midway through the procedure. The 2 RIP bands caused interference with the EIT data collection in some horses that sweated excessively (an example of mild interference is seen in Figure 1A,B in breath 3 of the saline measurement). In 1 horse, interference made EIT data analysis unusable. Both EIT and FP data sets were available in 17 horses.

**FIGURE 4:**
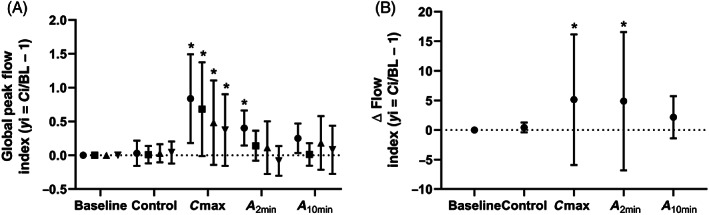
Mean (SD) global peak flow indices measured by electrical impedance tomography (EIT; • PIF_EIT_, ∎ PEF_EIT)_ and spirometry (▲ PIF_Spiro_, ▼ PEF_Spiro_; A) and the Δflow index (•) measured by flowmetric plethysmography (B) at baseline, after control nebulization (saline), at time of maximum histamine concentration when bronchoprovocation was deemed to have occurred (C_max_), 2 minutes post albuterol administration (*A*_2min_) and 10 minutes post albuterol (*A*_10min_). All variables were indexed for each horse as a fraction of baseline (*y*_i_ = *C*_i_/BL − 1; where *C*_i_ is the value measured with each examination point, respectively. * indicates values significantly different from the control nebulization

Horses showed significantly higher indexed EIT flow variables (PIF_EIT_: *P* < .001, PEF_EIT_: *P* < .001), indexed spirometry variables (PIF_Spiro_: *P* = .01, PEF_Spiro_: *P* = .02) and FP flow index, Δflow (*P* = .002) as well as respiratory rate (*P* < .001) at C_max_ compared to the diluent control nebulization (saline; Table 1). All values were lower at *A*_2min_ and were not significantly different from the control values at *A*_10min_ (Table 1). The global peak flow EIT indices PIF_EIT_ and PEF_EIT_ followed the same pattern of change as PIF_Spiro_ and PEF_Spiro_ as well as Δflow measured by flowmetric plethysmography (Figure 4).

**TABLE 1:** Mean (±SD) values for electrical impedance tomography (EIT), spirometry and flowmetric plethysmography (FP) for 17 horses challenged with the diluent control (saline), increasing histamine doses (4, 8, and 16 mg/mL), and subsequent albuterol administration (*A*_2min_ and *A*_10min_)

	Control (saline)	C_max_	*A*_2min_	*A*_10min_
PIF_EIT_ (AU)	1.86 (0.55)	3.20 (1.99)^a^	2.50 (0.89)^a^	2.22 (0.65)
PEF_EIT_ (AU)	2.56 (0.87)	4.09 (1.72)^a^	2.87 (1.16)	2.54 (1.04)
FV_slope_	0.97 (0.47)	1.47 (0.55)^a^	1.22(0.47)	1.15(0.60)
FV_slope_max	2.53 (0.87)	3.23 (1.12)	2.74 (0.90)	2.59 (0.92)
FV_intercept_/VT _EIT_	54.22 (12.25)	37.58 (14.32)^a^	47.72 (12.95)	52.57 (13.78)
VT_EIT_ (AU)	5.14 (0.91)	5.03 (1.21)	4.87(1.77)	4.76 (1.01)
Respiratory rate (bpm)	8.83 (2.68)	15.60 (7.10)^a^	12.25 (4.34)	10.48 (4.34)
PIF_Spiro_ (L/s)	3.57 (1.01)	4.97 (2.10)^a^	3.82 (1.57)	3.84 (1.42)
PEF_Spiro_ (L/s)	4.04 (1.79)	5.56 (3.11)^a^	3.90 (2.73)	3.62 (2.72)
Δflow	0.76 (0.60)	1.97 (1.26)^a^	1.69 (1.17)^a^	1.68 (1.01)
VT_Spiro_ (L)	6.23 (1.62)	6.18 (2.17)	5.60 (2.23)	6.56 (1.93)

*Notes*: FV_intercept_/VT_EIT_, the intercept between the steep and the horizontal part of the expiratory FV EIT; FV_slope_, global steepness of the slope of the initial expired volume of the expiratory EIT FV loop; FV_slope_max, the value of the steepest slope of all the FV loops generated within the ROI; PEF_EIT_, EIT peak global expiratory flow; PEF_Spiro_, pneumotachograph peak expiratory flow; PIF_EIT_, EIT peak global inspiratory flow; PIF_Spiro_, pneumotachograph peak inspiratory flow; VT_EIT_, EIT tidal volume; VT_Spiro_, pneumotachograph tidal volume.

^a^ Significant difference from the saline control.

Computing of FV loops was possible in 16/17 data sets. A biphasic flow pattern on the inspiratory part of the FV loop was observed after the administration of the control (n = 13), C_max_ (n = 2), *A*_2min_ (n = 3), and *A*_10min_ (n = 4). A biphasic flow pattern on the expiratory part of the FV loop was observed after the control nebulization (n = 16), C_max_ (n = 15), *A*_2min_ (n = 16), and *A*_10min_ (n = 15).

**FIGURE 5:**
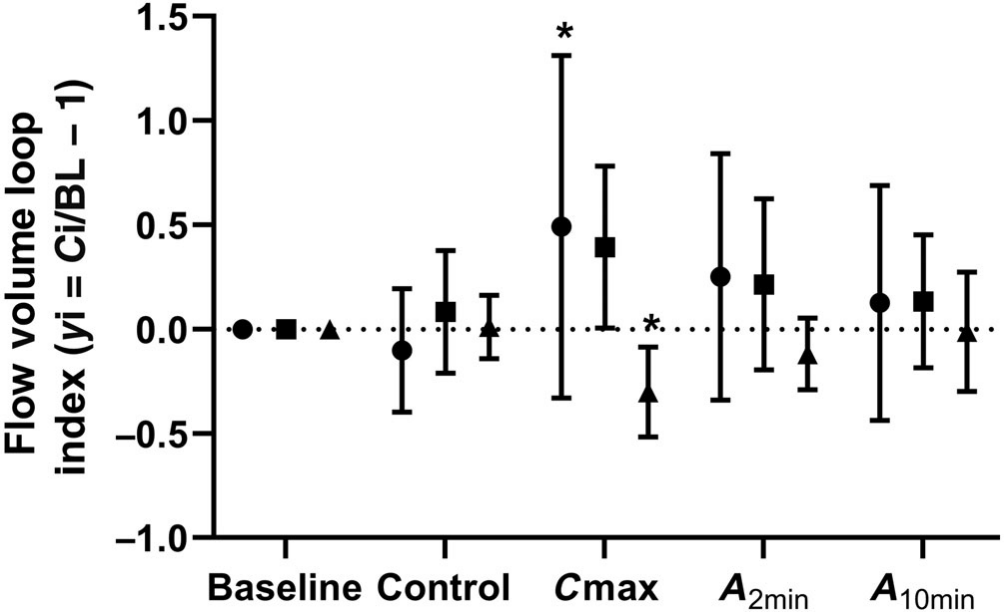
Graph showing the mean (SD) change in flow volume (FV) loop indices (• FV_slope_, ∎ FV_slope_max, and ▲ FV_intercept_/VT_EIT_) measured by electrical impedance tomography (EIT) at baseline, after administration of the control nebulization (saline), at time of maximum histamine concentration when bronchoprovocation was deemed to have occurred (C_max_), 2 minutes post albuterol administration (*A*_2min_) and 10 minutes post albuterol administration (*A*_10min_). All variables were indexed for each horse as a fraction of baseline (*y*_i_ = *C*_i_/BL − 1; where *C*_i_ is the value measured with each intervention respectively and saline is the value at saline measurement for the given variable. * indicates values significantly different from the control nebulization

The FV_slope_ at C_max_ was significantly higher (*P* = .03; Table 1) than at control nebulization but was not significantly different from control at *A*_2min_ and *A*_10min_ (Figure 5). Although FV_slope_max followed the same pattern, the change between the control nebulization and C_max_ was not significant. The FV_intercept_/VT _EIT_ was significantly lower (*P* < .001) at C_max_, but not significantly different from the control nebulization at *A*_2min_ and *A*_10min_ (Figure 5). The variables VT_EIT_ and VT_Spiro_ were not significantly different from the control nebulization values at any measurement point (Table 1).

Further analysis of all 5 EIT variables showed the variance in Δflow was best described by the variance in both flow variables, PIF_EIT_ and PEF_EIT_, with an adjusted *R* ^2^ of 0.51. When FV_slope_ was included, the adjusted *R* ^2^ increased to 0.53.

## 4 DISCUSSION

The aim of our study was to investigate whether histamine‐provoked changes in airflow and subsequent drug‐induced bronchodilatation can be detected using noninvasive EIT measurements, motivated by promising findings in a pilot study.^11^ Results showed global EIT peak flows can detect airflow changes during histamine challenge, and that EIT could detect a reversal of airflow changes induced by the administration of albuterol after histamine challenge. Additionally, novel variables (FV_slope_ and FV_intercept_/VT_EIT_) derived from the flow‐volume curve of the EIT signal were identified as also detecting airflow changes. Similar to the peak flows measured by spirometry and the primary FP variable, Δflow, these EIT‐derived flow indices significantly changed after histamine administration and returned to control values with subsequent albuterol administration.

Inhaled histamine has been shown to induce bronchoconstriction through activation of H1 receptors,^23^ and peak flows increase within the airways. ^11^, ^24^ This is true in both histamine‐induced bronchoconstriction and the natural disease state of severe asthma in horses.^25^, ^26^, ^27^, ^28^ Both devices, the EIT and the pneumotachograph, registered significantly higher measurements of inspiratory and expiratory peak flows during C_max_ (Figure 4). This result shows that global EIT flow variables, based on the assumption that the measured impedance change corresponds with inhaled and exhaled volume, follow the same pattern as airflow changes measured by a calibrated pneumotachograph during histamine challenge.

Our study showed that EIT also could detect airflow changes associated with bronchodilatation induced by albuterol. Albuterol has been shown to be effective in decreasing histamine induced contraction of equine bronchial muscle in vitro,^29^, ^30^ as well as being effective in inducing bronchodilatation in the natural disease state of severe asthma in horses.^31^, ^32^, ^33^, ^34^ This finding supports the use of EIT and its variables as a possible monitoring tool to assess treatment success in horses with asthma. Future studies on naturally occurring asthma in horses are necessary to explore this application.

To our knowledge, this study describes for the first time the computing of a FV loop from the EIT signal in veterinary medicine. The shape of the FV loop our study suggests a biphasic inspiratory and expiratory breathing pattern in most horses before the administration of histamine. This biphasic pattern also has been described in horses at rest using spirometry,^25^, ^26^, ^35^ and in combination with electromyography.^36^ During histamine challenge at C_max_, the biphasic pattern of the EIT‐derived FV loop was lost in all but 2 horses during inspiration, whereas a distinct biphasic pattern still was observed during expiration in all but 1 horse. Loss of the biphasic pattern of the FV loop in both inspiration and expiration has been described in horses with severe asthma.^25^, ^26^

The shape of FV loops with a high peak expiratory flow early in expiration followed by a low flow rate has been described in horses during severe airway obstruction.^26^ To quantify the expiratory flow pattern observed in the EIT‐derived FV loop, a global variable that described the shape of the global FV loop (FV_slope_) and 1 regional variable that identified the pixel with the steepest slope within the ROI and quantifies the steepness (FV_slope_ max) were designed (Figure 1C). At C_max_, the FV_slope_ was significantly higher than the control and subsequently returned to the control values after albuterol administration, suggesting that this variable can be used to describe changes in airflow in horses. The FV_slope_max followed the same pattern as FV_slope_, but the change was not significant. One reason for lack of significance was that both FV_slope_ and FV_slope_max displayed marked variability among horses. In some horses, the RIP bands of the FP unit, which applies a current traveling through the thoracic and abdominal bands, resulted in interference with the measured EIT signal, which influenced the plotting of the FV loop and made the computing of these 2 variables challenging for the software. The clinical usefulness of both EIT slope variables should be further explored in the absence of FP hardware.

The RIP bands used in FP have been used to assess changes in the breathing pattern of horses during airflow changes.^25^ Our intention was to create an EIT variable that identified these changes in breathing pattern as well. Similar to previous findings,^26^ we also observed an abrupt change in steepness of the expiratory curve slope in the FV loop with the slope becoming almost horizontal (Figure 2). The commencement of the horizontal part of the expiratory curve was subjectively synchronous with the activation of the abdominal muscles based on observation during the histamine challenge. This could be the component of the breathing cycle that maintains flow and decreases lung volume below the relaxed volume or functional residual capacity at the completion of passive exhalation, and forms the active component of the expiratory cycle as has been described in healthy horses.^36^ This change in flow was observed later in expiration during the histamine challenge when compared to baseline or after saline (control) nebulization. In an attempt to quantify this flow transition, a novel variable representing this point of change was created by calculating a ratio of between the intercept (between the steep and the horizontal part of the expiratory FV loop) and the tidal volume (FV_intercept_/VT _EIT_; Figures 2 and 1D). This variable was significantly lower after administration of histamine and subsequently returned to the control values with albuterol administration.

The primary FP variable, Δflow, which is a combined variable of airflow and thoracic volume changes,^18^ also was higher at C_max_, which confirmed the findings of the pilot study.^11^ One aim of our study was to evaluate which EIT variables best explain Δflow.^18^, ^22^ Multivariable analysis suggested that 51% of the variance in Δflow was explained by a combination of PIF_EIT_ and PEF_EIT_. This finding was anticipated because Δflow is calculated in part using airway flow measured by the pneumotachograph of the FP unit.^22^ The FV_slope_ also made a small contribution to the variance in Δflow.

As expected tidal volume did not change significantly with a change in flow variables with either measuring device during the study period. Previous studies have identified unchanging tidal volumes during bronchoconstriction in horses.^11^, ^25^, ^26^ The respiratory rate increased with histamine and then decreased with albuterol, whereas tidal volume did not change significantly, indicating an increase and then subsequent decrease in minute ventilation. The observed increase in respiratory rate results in a decrease in inspiratory^24^, ^25^ and expiratory time,^24^ which leads to the measured increase in flow variables at stable tidal volume. It has been postulated that the increase in minute ventilation could occur in response to mild hypoxemia as a consequence of airway narrowing.^28^

It remains challenging to assess pulmonary mechanics using existing pulmonary function testing modalities immediately after exercise in the horse. The minimal portable EIT instrumentation which can be powered by a battery, is noninvasive and does not require a face mask is well suited to clinical situations. It is possible that EIT instrumentation can be undertaken in the field, allowing airway status to be investigated before and immediately after exercise in horses suffering from naturally‐occurring asthma.^37^ Given that the EIT can detect both narrowing and subsequent widening of airways, it is plausible that EIT will be able to determine the presence of exercise‐induced bronchoconstriction associated with mild asthma immediately after exercise and test the effects of treatment. Furthermore, the continued development of novel EIT variables may allow for subtle changes in airway function that are performance limiting but not clinically applicable at rest to be more accurately assessed.

A detomidine continuous rate infusion was used in our study because sedation was required for collection of the data that demanded a tight fitting facemask over an extend time period. The continuous rate infusion was started immediately after the loading bolus of detomidine and before instrumentation. The infusion rate was not changed throughout the study period. This guaranteed a steady state of sedation and pharmacological parameters for the duration of the histamine nebulization and subsequent albuterol administration.^38^ It also ruled out the possible bronchodilatatory effects of detomidine.^39^

A limitation of our study was occasional interference between the FP instrumentation and the EIT instrumentation because both emit an electrical signal. This interference was unpredictable and generally could be filtered out in the postprocessing phase. The variable most affected by interference was the FV_slope_. Furthermore, movement artifacts can make EIT evaluation difficult, and different length of belts might be required to guarantee good electrode‐skin contact in various sizes of horses. The horses in our study were sedated and movement artifacts therefore were neglectable. We used a custom‐made neoprene electrode belt that fitted all of the horses and allowed good skin‐electrode contact during all measurements without the need to clip hair.

Another limitation is that albuterol was administered as puffs into the mask, which makes precise dosing of the drug impossible. The aim of albuterol administration was to show a gradual improvement of EIT values back to baseline, which made early measurement after drug administration necessary. To meet this aim, this method of application for the albuterol was chosen because it was essential to leave the mask in place to be able to start data recording immediately. Finally, healthy horses were used in our study, further studies to validate EIT using a naturally‐occurring airway obstruction model against other methods of pulmonary function testing will be required to support the use of this modality as a diagnostic tool for horses with asthma.

## 5 CONCLUSION

The EIT variables, PIF_EIT_ and PEF_EIT_, and the variables derived from the FV loops, FV_slope_ and FV_intercept_/VT _EIT_, detected changes in airflow after histamine bronchoprovocation in horses and subsequent albuterol administration. Global EIT flow variables showed the same pattern of change as did flow variables measured by spirometry and best explained Δflow. This modality could be useful to detect changes in airflow in horses with asthma and further work is required to determine if a combination of the variables measured by the EIT may be useful to detect subtle changes in pulmonary function in horses with asthma.

## CONFLICT OF INTEREST DECLARATION

Andy Adler and Martina Mosing are developing a commercial product that uses technology developed in this paper. All other authors have declared that their contribution to the research was conducted in the absence of any commercial or financial relationships that could be construed as a potential conflict of interest.

## OFF‐LABEL ANTIMICROBIAL DECLARATION

Authors declare no off‐label use of antimicrobials.

## INSTITUTIONAL ANIMAL CARE AND USE COMMITTEE (IACUC) OR OTHER APPROVAL DECLARATION

This experimental in vivo study was performed on experimental horses and was approved by Murdoch University Institutional Animal Ethics committee (permit number R2895/17).

## HUMAN ETHICS APPROVAL DECLARATION

Authors declare human ethics approval was not needed for this study.

## References

[1] Couetil LL , Cardwell JM , Gerber V , et al. Inflammatory airway disease of horses—revised consensus statement. J Vet Intern Med. 2016;30:503‐515. 26806374 10.1111/jvim.13824 PMC4913592

[2] Couetil L , Cardwell J , Leguillette R , et al. Equine asthma: current understanding and future directions. Front Vet Sci. 2020;7:450. 10.3389/fvets.2020.00450. 32903600 PMC7438831

[3] Kinnison T , Cardwell JM . Conflict between direct experience and research‐based evidence is a key challenge to evidence‐based respiratory medicine on British racing yards. Front Vet Sci. 2020;7:266. 10.3389/fvets.2020.00266. 32537459 PMC7267464

[4] Ambrisko TD , Schramel JP , Adler A , et al. Assessment of distribution of ventilation by electrical impedance tomography in standing horses. Physiol Meas. 2016;37:175‐186. 26711858 10.1088/0967-3334/37/2/175

[5] Mosing M , Auer U , MacFarlane P , et al. Regional ventilation distribution and dead space in anaesthetized horses treated with and without continuous positive airway pressure: novel insights by electrical impedance tomography and volumetric capnography. Vet Anaesth Analg. 2018;45:31‐40. 29222030 10.1016/j.vaa.2017.06.004

[6] Mosing M , Waldmann AD , MacFarlane P , et al. Horses auto‐recruit their lungs by inspiratory breath holding following recovery from general anaesthesia. PLoS One. 2016;11:e0158080. 27331910 10.1371/journal.pone.0158080 PMC4917253

[7] Mosing M , Waldmann AD , Raisis A , et al. Monitoring of tidal ventilation by electrical impedance tomography in anaesthetised horses. Equine Vet J. 2019;51:222‐226. 30035329 10.1111/evj.12998

[8] Adler A , Amyot R , Guardo R , et al. Monitoring changes in lung air and liquid volumes with electrical impedance tomography. J Appl Physiol. 1997;83:1762‐1767. 9375349 10.1152/jappl.1997.83.5.1762

[9] Frerichs I , Hahn G , Hellige G . Thoracic electrical impedance tomographic measurements during volume controlled ventilation‐effects of tidal volume and positive end‐expiratory pressure. IEEE Trans Med Imaging. 1999;18:764‐773. 10571381 10.1109/42.802754

[10] Bodenstein M , Boehme S , Bierschock S , et al. Determination of respiratory gas flow by electrical impedance tomography in an animal model of mechanical ventilation. BMC Pulm Med. 2014;14:73. 24779960 10.1186/1471-2466-14-73 PMC4012093

[11] Secombe C , Waldmann AD , Hosgood G , et al. Evaluation of histamine‐provoked changes in airflow using electrical impedance tomography in horses. Equine Vet J. 2020;52:556‐563. 31793056 10.1111/evj.13216

[12] Henneke DR , Potter GD , Kreider JL , et al. Relationship between condition score, physical measurements and body fat percentage in mares. Equine Vet J. 1983;15:371‐372. 6641685 10.1111/j.2042-3306.1983.tb01826.x

[13] Adler A , Arnold JH , Bayford R , et al. GREIT: a unified approach to 2D linear EIT reconstruction of lung images. Physiol Meas. 2009;30:S35. 19491438 10.1088/0967-3334/30/6/S03

[14] Costa ELV , Chaves CN , Gomes S , et al. Real‐time detection of pneumothorax using electrical impedance tomography. Crit Care Med. 2008;36:1230‐1238. 18379250 10.1097/CCM.0b013e31816a0380

[15] Frerichs I , Amato MBP , Van Kaam AH , et al. Chest electrical impedance tomography examination, data analysis, terminology, clinical use and recommendations: consensus statement of the TRanslational EIT developmeNt stuDy group. Thorax. 2017;72:83‐93. 27596161 10.1136/thoraxjnl-2016-208357 PMC5329047

[16] Karagiannidis C , Waldmann AD , Róka PL , et al. Regional expiratory time constants in severe respiratory failure estimated by electrical impedance tomography: a feasibility study. Crit Care. 2018;22:221. 30236123 10.1186/s13054-018-2137-3 PMC6148957

[17] Ambulatory Monitoring, I . The Open Pleth™ System Manual: Version 3.3 . New York. 2010:1–60.

[18] Hoffman AM . Clinical application of pulmonary function testing in horses. In: Lekeux P , ed. Equine Respiratory Diseases Ithaca. USA: International Veterinary Information Service; 2002:1‐29.

[19] Róka PL , Waldmann AD , Böhm SH , et al. Software tool for analyzing ventilation EIT data. In: 16th International Conference on Biomedical Applications of electrical impedance tomography; 2016.

[20] Stowe S , Mosing M , Adler A . Software to automate functional analysis of reconstructed EIT data. In: 20th International Conference on Biomedical Applications of Electrical Impedance Tomography; 2020.

[21] Adler A , Lionheart WRB . Uses and abuses of EIDORS: an extensible software base for EIT. Physiol Meas. 2006;27:S25. 16636416 10.1088/0967-3334/27/5/S03

[22] Hoffman A , Kuehn H , Riedelberger K , et al. Flowmetric comparison of respiratory inductance plethysmography and pneumotachography in horses. J Appl Physiol. 2001;91:2767‐2775. 11717245 10.1152/jappl.2001.91.6.2767

[23] Yamauchi K , Ogasawara M . The role of histamine in the pathophysiology of asthma and the clinical efficacy of antihistamines in asthma therapy. Int Mol Sci. 2019;20:1733. 10.3390/ijms20071733 PMC6480561 30965592

[24] Guthrie AJ , Beadle RE , Bateman RD , White CE . The effects of three models of airway disease on tidal breathing flow‐volume loops of thoroughbred horses. Vet Res Commun. 1995;19:517‐527. 8619290 10.1007/BF01839340

[25] Hoffman AM , Oura TJ , Riedelberger KJ , et al. Plethysmographic comparison of breathing pattern in heaves (recurrent airway obstruction) versus experimental bronchoconstriction or hyperpnea in horses. J Vet Intern Med. 2007;21:184‐192. 17338167 10.1892/0891-6640(2007)21[184:pcobpi]2.0.co;2

[26] Petsche VM , Derksen FJ , Robinson NE . Tidal breathing flow‐volume loops in horses with recurrent airway obstruction (heaves). Am J Vet Res. 1994;55:885‐891. 7978623

[27] Herholz C , Straub R , Braendlin C , et al. Measurement of tidal breathing flow‐volume loop indices in horses used for different sporting purposes with and without recurrent airway obstruction. Vet Rec. 2003;152:288‐292. 12650471 10.1136/vr.152.10.288

[28] Robinson NE , Olszewski MA , Boehler D , et al. Relationship between clinical signs and lung function in horses with recurrent airway obstruction (heaves) during a bronchodilator trial. Equine Vet J. 2000;32:393‐400. 11037260 10.2746/042516400777591147

[29] Pozzoli C , Bertini S , Poli E , et al. Relaxing effects of clenbuterol, ritodrine, salbutamol and fenoterol on the contractions of horse isolated bronchi induced by different stimuli. Res Vet Sci. 2020;128:43‐48. 31710963 10.1016/j.rvsc.2019.10.022

[30] Matera MG , Calzetta L , Rogliani P , et al. Evaluation of the effects of the R‐ and S‐enantiomers of salbutamol on equine isolated bronchi. Pulm Pharm Ther. 2011;24:221‐226. 10.1016/j.pupt.2010.12.008 21195788

[31] Tanquerel L , Fillion‐Bertrand G , Lavoie J‐P , et al. Effects of magnesium sulfate infusion on clinical signs and lung function of horses with severe asthma. Am J Vet Res. 2018;79:664‐673. 30085859 10.2460/ajvr.79.6.664

[32] Bertin FR , Ivester KM , Couetil LL . Comparative efficacy of inhaled albuterol between two hand‐held delivery devices in horses with recurrent airway obstruction. Equine Vet J. 2011;43:393‐398. 21496081 10.1111/j.2042-3306.2010.00313.x

[33] Derksen FJ , Olszewski MA , Robinson NE , et al. Aerosolized albuterol sulfate used as a bronchodilator in horses with recurrent airway obstruction. Am J Vet Res. 1999;60:689‐693. 10376893

[34] Arroyo MG , Couëtil LL , Nogradi N , et al. Efficacy of inhaled Levalbuterol compared to albuterol in horses with recurrent airway obstruction. J Vet Intern Med. 2016;30:1333‐1337. 27282625 10.1111/jvim.14320 PMC5089594

[35] Gillespie JR , Tyler WS , Eberly VE . Pulmonary ventilation and resistance in emphysematous and control horses. J Appl Physiol. 1966;21:416‐422. 5949047 10.1152/jappl.1966.21.2.416

[36] Koterba A , Kosch PC , Beech J , et al. Breathing strategy of the adult horse (*Equus caballus*) at rest. J Appl Physiol. 1988;64:337‐346. 3356653 10.1152/jappl.1988.64.1.337

[37] Herteman N , Mosing M , Waldmann A , et al. Exercise‐induced airflow changes in horses with equine asthma measured by electrical impedance tomography(EIT) J Vet Intern Med. 2021 [submitted for publication]. 10.1111/jvim.16260 PMC8478024 34505734

[38] Gozalo‐Marcilla M , de Oliveira AR , Fonseca MW , et al. Sedative and antinociceptive effects of different detomidine constant rate infusions, with or without methadone in standing horses. Equine Vet J. 2019;51:530‐536. 30485499 10.1111/evj.13054

[39] Menozzi A , Pozzoli C , Poli E , et al. Effects of selective α2 ‐adrenergic receptor agonists on electrical field‐stimulated contractions of isolated bronchi in horses. J Vet Pharmacol Ther. 2018;41:246‐253. 29164631 10.1111/jvp.12470

